# Impact of vaccination on invasive pneumococcal disease in Italy 2007–2017: surveillance challenges and epidemiological changes

**DOI:** 10.1017/S0950268820001077

**Published:** 2020-05-18

**Authors:** R. Monali, E. De Vita, F. Mariottini, G. Privitera, P. L. Lopalco, L. Tavoschi

**Affiliations:** Department of Translational Research and New Technologies in Medicine and Surgery, University of Pisa, Pisa, Italy

**Keywords:** Pneumococcal infection, surveillance, vaccination (immunisation)

## Abstract

Surveillance of new cases of invasive pneumococcal disease (IPD) in Italy was started in 2007 by the Ministry of Health (MoH). In 2012, pneumococcal childhood vaccination was introduced at the national level and, in 2017, for citizens aged 65 years and over. We describe here IPD epidemiology in Italy over the past 10 years investigating the impact of the vaccine programme on disease burden. Reports of IPD cases, data on serotype and vaccination coverage (VC) data were obtained from MoH annual reports, for the period 2007–2017. IPD notification rate and proportion by year, region, age and serotype were calculated. In 2007, 525 cases were reported (rate 0.88/100 000), rising to 1703 cases (rate 2.82/100 000) in 2017. The distribution of IPD cases by age group over time registered the largest share among individuals aged 65 years and over. A decreasing trend in notification rate was observed among those aged 0–4 years. During the same period, the 24-month VC increased, ranging from 80.9% to 96.7% in 2017. Molecular data indicated re-emergence of PPSV23-specific serotypes and non-vaccine serotypes. We observed an increase in IPD notifications during 2007–2017, likely due to an improved surveillance system, at least in some regions, with the relative quota of IPD notifications decreasing among vaccinated children cohorts. Further strengthening of IPD surveillance system, including molecular and vaccine coverage data, would be needed to assess and inform pneumococcal vaccination strategies in Italy.

## Introduction

*Streptococcus pneumoniae* is one of the most important causes of morbidity and mortality worldwide, causing 1.6 million deaths annually [1]. It is the causative agent of several clinical syndromes both non-invasive and invasive. Invasive pneumococcal disease (IPD) is determined when the micro-organism penetrates and invades normally sterile sites such as meninges and the bloodstream [2]. The burden of IPD is high, especially among infants younger than 2 years of age and older adults who have the highest notification and case fatality rate (10–25%), despite the availability of effective antibiotic therapy [3].

Occurrence of IPD is influenced by several factors including individual immune status [2], presence of comorbidities such as chronic cardiovascular, hepatic or pulmonary diseases, diabetes mellitus [3], smoking status, environmental and pathogen-related factors. More than 90 different serotypes of *S. pneumoniae* have been identified, with various level of virulence [2].

Effective pneumococcal vaccine formulations are available, including 7-valent (PCV7), 10-valent (PCV10), 13-valent (PCV13) conjugate vaccines and 23-valent (PPSV23) non-conjugate vaccine [4]. Their introduction as part of national immunisation schedules contributed to reducing IPD notifications and mortality caused by IPD in Europe [5].

In Italy, the population access to pneumococcal immunisation is regulated by the National Plan for Vaccine Prevention (NPVP), however with some variability at the sub-national level due to a certain degree of regional autonomy in the organisation of health services [6]. The implementation of the national plan is monitored as part of the essential levels of public health care [6]. In Italy, the first pneumococcal vaccine (PCV7), containing serotypes 4, 6B, 9V, 14, 18C, 19F and 23F, has been introduced in 2001 [7], and between 2006 and 2010, all 20 regions recommended or introduced PCV7 in their childhood immunisation schedule [8]. The PCV10 vaccine, containing serotypes 1, 5 and 7F in addition to those contained in PCV7, has been authorised for marketing in 2009. However, the PCV10 vaccine, despite its availability, was little used because almost at the same time (April 2010) PCV13 was licensed.

In 2010, the Ministry of Health (MoH) issued a recommendation to replace PCV7 with PCV13, containing six additional serotypes (1, 3, 5, 6A, 7F, 19A) [9], and the following NPVP 2012–2014 included active and free offer of three PCV13 doses for newborns at 3, 5–6 and 11–13 months of age and a catch-up for those vaccinated with PCV7. The plan set to achieve and maintain PCV13 vaccination coverage (VC) of at least 95% in children <24 months of age [9, 10]. PPSV23 has been authorised for the first time in Italy on 3 May 2000. During the latest years, PPSV23 vaccination has been offered to people 65 years of age and over in several Italian regions. The latest NPVP (2017–2019) complements the vaccination offer and make it homogeneous at the national level with the provision of free anti-pneumococcal vaccination in subjects of 65 years of age [6], in line with other high-income countries that have introduced this additional intervention in the past years [11].

The surveillance of IPD was introduced in Europe in 2010, and varies markedly across the countries, including differences in laboratory methods to confirm cases, reporting protocols and medical practices. As a result, rates are heterogeneous across the region, varying from less than 1 to more than 10 cases per 100 000 population [5], possibly masking true differences in the incidence. In addition, longitudinal trends of IPD notification rates varied over the period 2010–2017 with some recognisable temporal and geographical patterns. The introduction of large-scale vaccination and heterogeneity in the surveillance systems may explain some of the observed changes. Weaknesses in the surveillance system are not solely responsible for this heterogeneity of rates, in fact other factors such as seasonal and annual fluctuations, intercurrent viral infection, recent use of antibiotics in the population can play a role.

While some countries such as Ireland and UK [12] showed a stable trend, others, such as Hungary [13] and Italy [14], showed an increasing rate of reported cases over time. Finally, few countries, including Denmark and France [12], were characterised by a declining rate.

When assessed against the backdrop of the European region, Italy is a low IPD notification country. However, remarkable differences may be observed across regions [15]. Enhanced IPD surveillance is implemented in Italy since March 2007, when meningitis monitoring was added to the other invasive disease manifestations, and is coordinated by the MoH [16]. The existing surveillance system aims at describing and monitoring the epidemiology of IPD, including the distribution of circulating serogroups/serotypes and at assessing the impact of vaccination programmes. The system relies on case-based notifications by physicians following microbiological confirmation of the diagnosis, collated at the regional level and transmitted monthly to the MoH [17].

Here, we collated available epidemiological and microbiological data collected since the roll-out of enhanced IPD surveillance in Italy in order to describe the epidemiology of disease in the context of increasing VC in the country.

## Materials and methods

Epidemiological data on notifications of IPD cases and serotype characterisation were sourced from the annual reports of the surveillance system for invasive meningococcal, pneumococcal and haemophilic bacterial diseases and bacterial meningitis, and publicly available on the National Health Institute (ISS) website for the years 2007–2017 [14]. Surveillance of bacterial diseases includes case definitions based on laboratory criteria [14]. For IPD surveillance, each case must be confirmed by one of the following laboratory criteria, which is based on the European case definition: isolation of *S. pneumoniae* from a normally sterile site; detection of the presence of *S. pneumoniae* nucleic acid in a normally sterile site or detection of *S. pneumoniae* antigens in a normally sterile site (except blood). Suspected but unconfirmed cases are not reported in the system [15]. The ISS National Reference Laboratory offers technical support to peripheral laboratories in the country for diagnosis confirmation and serotyping [15].

The population data were sourced from the National Institute for Statistics (ISTAT) for the years 2007–2017 and for each Italian region. The population was defined as all resident individuals at the 31st of December of the reference year, to reflect the latest yearly ISTAT updates.

VC data for the period 2007–2017 were sourced from the MoH website. VC data for 100 000 inhabitants at 24 months are submitted yearly to the MoH by each region. The 24-month VC is defined as the proportion of children belonging to a given birth cohort fully vaccinated at the 31st of December of 2 years after the year of birth.

We analysed IPD notifications by year of report, region of residence and age. We calculated IPD notification rates by region and by age and annual proportion of IPD notifications by age group. We classified the Italian region according to the reported IPD notification rate, using a cut-off of 1 case per 100 000 inhabitants as proposed elsewhere [17]. Finally, the distribution of *S. pneumoniae* serotypes was analysed according to their inclusion in vaccine formulations (PCV7, PCV 10, PCV13 and PPSV23) during the study period.

We generated the map using the EMMa software, created by the European Centre for Disease Prevention and Control. All analyses were performed using Microsoft Excel.

## Results

Between 2007 and 2017, a total of 10 791 cases of IPD were notified in Italy, with an overall increasing trend. In 2007, a total of 524 cases were reported by 20 regions corresponding to a notification rate of 0.88 per 100 000 population. In 2017, a total of 1703 IPD cases were reported to the Italian MoH by 20 regions corresponding to an overall notification rate of 2.82 per 100 000 inhabitants. In 2017, the IPD age-specific notification rates were: 5.7 per 100 000 infants under 1 year of age; 2.2 per 100 000 children aged 1–4 years; 1.8 per 100 000 individuals aged between 25 and 64 years and 7.3 among those aged above 64 years. Subjects with age <1 and >64 years showed the highest notification rates. Age classes with a rate <1 per 100 000 were 5–9, 10–14 and 15–24 years, with a value of 0.4–0.5 and 0.4 per 100 000, respectively.

**Fig. 1.**
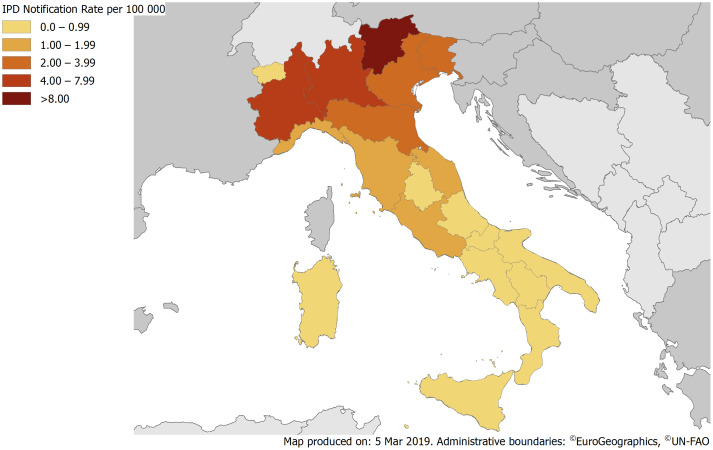
IPD notification rates per 100 000 population by region, Italy, 2017.

In 2017, the notification rates of IPD for 100 000 inhabitants varied widely across Italian regions from 0 to 8.7 per 100 000. Distribution of notification rates across Italy showed a marked North-South geographical gradient and were: 0.4 in Abruzzo; 0.8 in Aosta Valley; 0.4 in Apulia; 0.5 in Basilicata; 0.5 in Calabria; 0.8 in Campania; 3.7 in Emilia-Romagna; 3.7 in Friuli-Venezia Giulia; 1.4 in Lazio; 1.8 in Liguria; 6.6 in Lombardy; 1.0 in Marche; 0.6 in Molise; 7.1 in Piedmont; 0.7 in Sardinia; 0.3 in Sicily; 8.7 in Trentino Alto-Adige; 1.5 in Tuscany; 0.0 in Umbria; 2.7 in Veneto (Fig. 1).

**Fig. 2.**
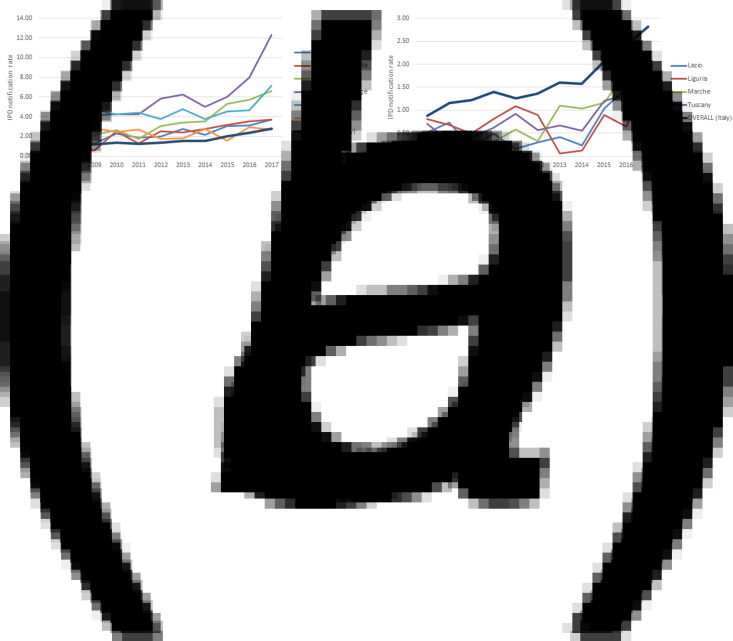
IPD notification rates per 100 000 population, selected region groups, Italy, 2007–2017. (a, left panel) Regions of group 1; (b, right panel) regions of group 2.

Regional variability was investigated further by dividing regions into sub-groups based on the regional IPD notification rates over time, and the findings are presented in Figure 2. On the left of the panel are shown (Fig. 2a) IPD notification rates reported by the northern regions, namely Emilia-Romagna, Friuli-Venezia Giulia, Lombardy, Trentino-Alto Adige, Piedmont, Veneto (Group 1) from 2007 to 2017. These regions reported a notification rate equal to or above the threshold of 1 per 100 000 population during the study period with an increasing trend: from 2.2 in 2007 to 3.7 in 2017 in Emilia Romagna, from 1.1 to 3.7 in Friuli Venezia Giulia, from 1.5 to 6.6 in Lombardy, from 1.3 to 8.7 in Trentino Alto Adige, from 2.2 to 2.7 in Veneto, from 1.0 to 7.1 in Piedmont. On the right of the panel (Fig. 2b), IPD notification rates reported mainly by central Italian regions (Lazio, Marche, Tuscany and Liguria) are presented (Group 2). These regions were characterised by a notification rate below 1 per 100 000 threshold in 2007, which gradually increased above the threshold: from 0.5 in 2007 to 1.4 in 2017 in Lazio, from 0.8 to 1.8 in Liguria, from 0.2 to 1.0 in Marche, from 0.7 to 1.5 in Tuscany. The remaining regions (Abruzzo, Apulia, Calabria, Campania, Basilicata, Molise, Sardinia, Sicily, Umbria) are below the threshold throughout the study period. Notification rates for specific age groups in each region were not available.

**Fig. 3.**
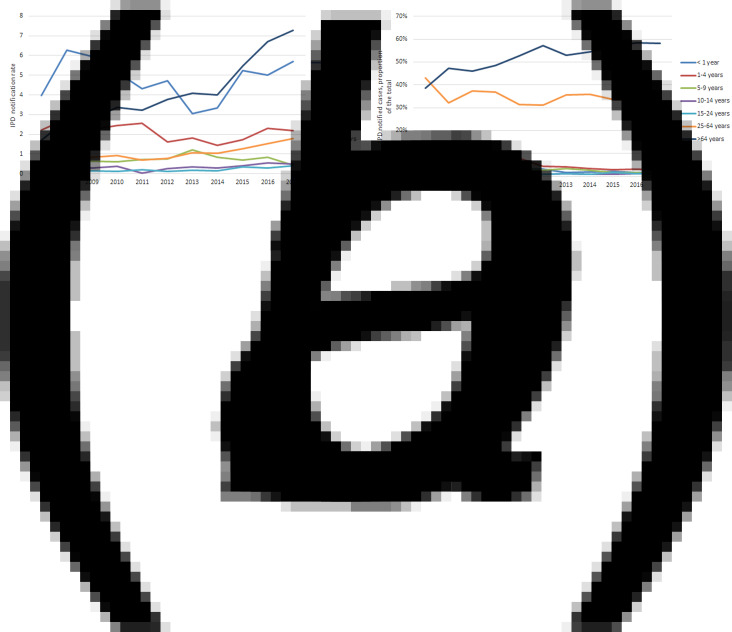
IPD notifications by age groups, Italy, 2007–2017. IPD notification rates per 100 000 individuals by age group (a), relative proportion of IPD cases by age (b).

In the period 2007–2017 in Italy, a total of 10 779 (99.9%) cases were notified with information on age. Age-specific IPD notification rates showed a linear increase during the period 2007–2017 for the following age groups: 5–9, 10–14, 15–24, 25–64 and >64 years, with the largest increase recorded for the group >64 years old (Fig. 3a). The notification trends for age groups <1 and 1–4 showed a non-linear trend with a decrease until 2013 and increasing after that. To adjust for changes that may have occurred in the regional surveillance systems, we investigated further trends over time in the different age groups, assuming no underlying differences in physicians’ ascertainment and reporting practices (i.e. expanded testing/reporting for one age group over another). We expressed age-specific notified cases as a relative proportion of the total (Fig. 3b). During the period 2007–2017, IPD notifications increased the most among individuals aged above 64 years as compared to all other age groups. The proportion of new cases among children aged <14 years showed a steady decreasing trend over the study period.

VC data were not available for all regions across the study period (2007–2017) (Table 1). The number of regions with non-available coverage data has almost halved (from 10 to 6) in 2012 compared to 2007. Since 2012 national coverage data were available, increasing from 81.2% to 90.9% in 2017. In 2017, reported VC showed some variability across regions. In particular, it was >90% in 16 regions (Abruzzo, Aosta Valley, Apulia, Basilicata, Calabria, Emilia Romagna, Lazio, Liguria, Lombardy, Marche, Molise, Trentino (Trento), Piedmont, Sardinia, Tuscany, Umbria) and >95% in Basilicata, Molise and Sardinia (96.7%, 96.0% and 95.4%, respectively), and it was >80% in the remainder. With the exception of Liguria and Umbria, where there is a drop in VC following 2012 (the first year with available data), an increasing trend in VC is noted in all the other regions during the period 2007–2017. No data were available on VC among adults.

**Table 1.** Reported vaccination coverage for pneumococcal vaccine at 24-month of age, by year and by region, Italy, 2007–2017

	Year of report										
	2007	2008	2009	2010	2011	2012	20131	2014	2015	2016	2017
Region											
Abruzzo	n.d	n.d	n.d	n.d	n.d	53.7	56.1	83.6	86.3	89.3	91.1
Aosta Valley	n.d.	n.d.	85.4	89.7	90.4	88.8	89.7	84.4	88.5	87.4	91.3
Apulia	75.7	80.4	85.4	86.3	82.8	93.1	92.9	93.5	92.5	91.4	92.0
Basilicata	92.7	92.7	96.3	97.3	98.5	98.6	98.7	98.5	97.1	97.0	96.7
Calabria	57.1	68.1	75.3	81.4	86.9	43.6	44.5	92.0	88.7	90.0	94.6
Campania	n.d.	n.d.	n.d.	n.d.	n.d.	n.d.	n.d.	76.6	83.0	82.1	88.2
Emilia Romagna	43.3	n.d.	94.3	94.6	94.1	n.d.	94.1	92.5	91.5	90.6	92.7
Friuli Venezia Giulia	n.d.	n.d.	n.d.	n.d.	74.9	83.6	86.5	82.4	81.0	81.4	83.8
Lazio	n.d.	n.d.	n.d.	n.d.	n.d.	n.d.	89.2	91.3	91.9	93.8	92.3
Liguria	n.d.	n.d.	n.d.	n.d.	n.d.	94.3	93.7	92.2	92.8	91.8	93.0
Lombardy	35.9	46.9	58.5	66.1	71.7	77.4	83.7	79.4	86.8	85.7	92.5
Marche	24.2	35.5	45.5	59.7	65.8	88.3	93.4	89.7	88.0	89.4	90.7
Molise	n.d.	n.d.	n.d.	n.d.	n.d.	53.5	96.0	94.6	92.6	91.5	96.0
Trentino (Bolzano)	14.3	23.6	40.2	72.6	75.5	71.2	78.8	80.7	81.7	80.5	80.9
Trentino (Trento)	37.7	69.5	85.1	84.0	84.6	85.7	87.6	87.1	87.3	89.2	90.9
Piedmont	10.8	19.3	27.8	29.1	44.7	n.d.	92.7	92.5	91.3	91.8	92.8
Sardinia	n.d.	n.d.	n.d.	n.d.	n.d.	n.d.	64.8	95.0	94.1	94.2	95.4
Sicily	83.9	88.7	90.7	93.6	94.3	95.7	92.9	91.7	89.4	88.5	88.1
Tuscany	n.d.	n.d.	n.d.	88.2	93.5	n.d.	94.0	93.8	92.9	89.0	90.4
Umbria	n.d.	n.d.	n.d.	n.d.	n.d.	94.4	94.9	94.2	90.3	91.6	94.3
Veneto	83.7	86.1	87.5	88.3	78.2	89.7	88.4	85.6	84.6	84.5	86.6
All regions	n.d.	n.d.	n.d.	n.d.	n.d.	81.2	85.6	89.1	89.2	89.1	90.9

**Fig. 4.**
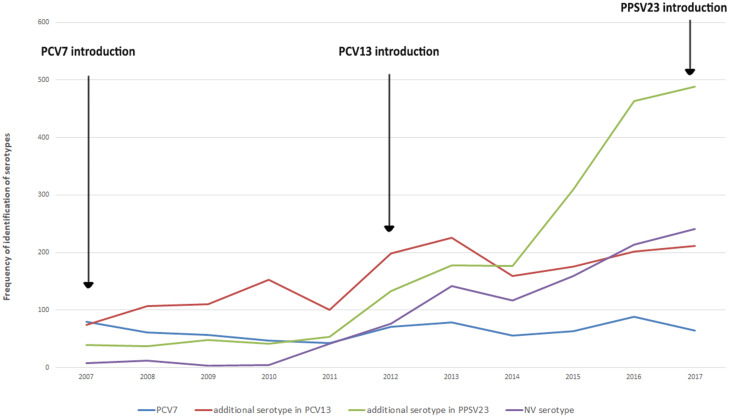
Frequency of identification of pneumococcal serotypes by vaccine formulation, Italy, 2007–2017.

Over the study period, serotyping data were available for 51.0% (5496) of the notified cases, with an increasing trend over time varying from 39.7% in 2007 to 59.2% in 2017. The relative proportion of *S. pneumoniae* serotypes contained in the PCV7 vaccine decreased slowly from 31.0% in 2007 to about 6.3% in 2017. Serotypes contained in the PCV10 vaccine varied from 13.2% in 2007 to 2.6% in 2017, with a peak of 27.7% in 2010, followed by a constant decrease (see online Supplementary materials). *S. pneumoniae* additional serotypes included in the PCV13 vaccine increased from 2007 to 2010 when they constituted the 25.3% of total and decreased after that to representing the 18.3% of the total in 2017. The proportion of the serotypes contained in the PPSV23 vaccine only increased gradually between 2007 (15.1%) and 2014, showing a steep rise afterwards (48.1%) (Fig. 4). Lastly, the relative share of *S. pneumoniae* serotypes not covered by any of the existing vaccine formulations showed an oscillating trend over the study period with similar figures reported in 2007 (25.2%) and 2017 (24.7%) (online Supplementary materials).

**Fig. 5.**
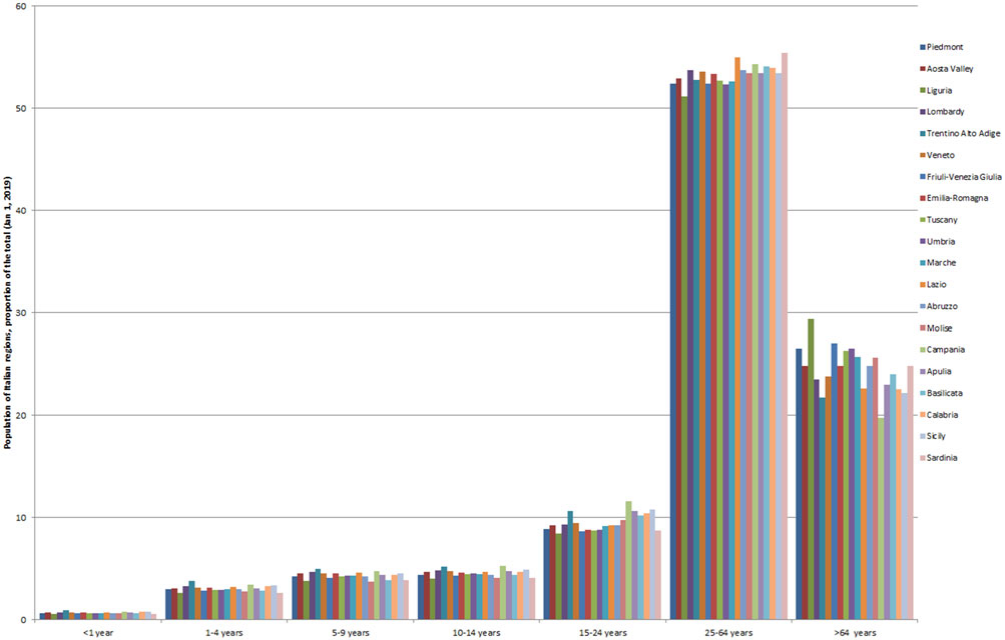
Italian population, by age and by region, the proportion of the total (Jan 1, 2019).

**Fig. 6.**
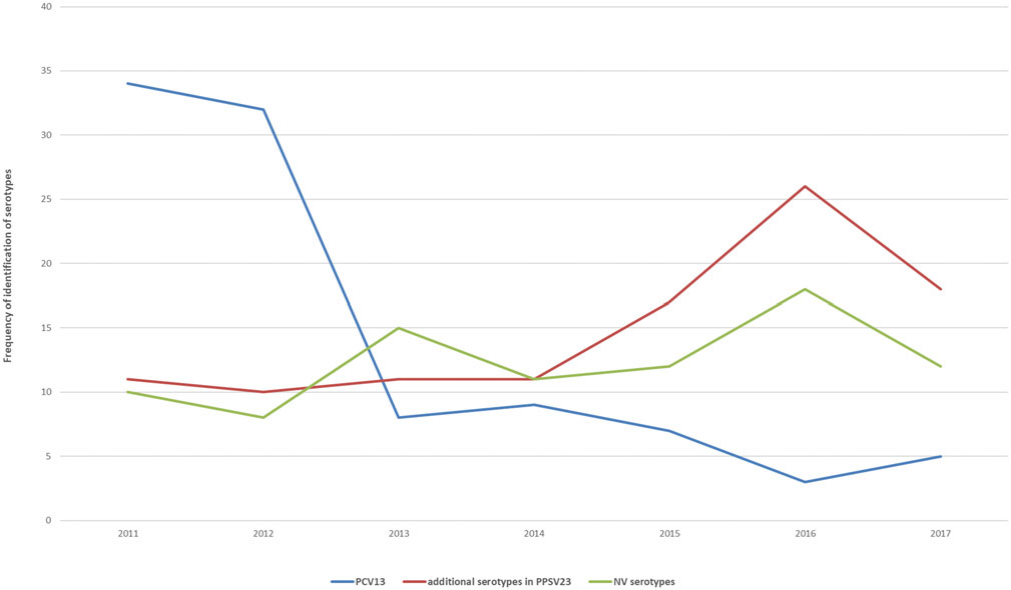
Frequency of identification of serotypes in IPD, Italy, 2007–2017.

**Fig. 7.**
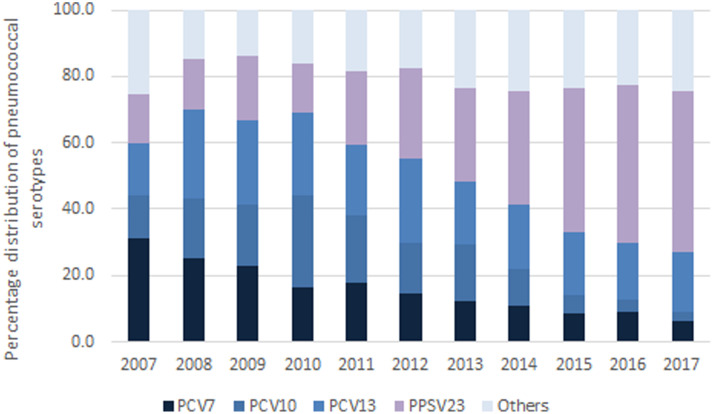
Frequency of identification of pneumococcal serotypes by vaccine formulation, Italy, 2007–2017.

The serotypes that have seen a greater increase during the whole period studied are 3 (contained in PCV13), 8, 12FF and 22F (contained in PPSV23). Comparing available age-specific data for the period 2011–2017 for the population aged 0–4 years and 2015–2017 for the population aged above 64 years, it emerged that 19A and 3 (contained in PCV13) are decreasing among infants and children, while 8 and 12F (contained in PPSV23) are common among adults and progressively increasing among children (see online Supplementary materials).

## Discussion

With this study, we provide a comprehensive overview of the epidemiology of IPD in Italy over more than a decade, collating all available data sources. While syndromic surveillance for invasive bacterial disease has been established in Italy in 1994, it is only from 2007 that standardised reporting of aetiological agents has been introduced [2].

According to our findings, between 2007 and 2017, almost 10 800 IPD cases were notified, with an overall increasing trend and a high heterogeneity across the country. IPD notification rate increased the most among people aged 65 years and over. Childhood VC data were available for most of the regions throughout the period and showed an increasing trend with national coverage peaking in 2017 at 90.9%. Microbiological surveillance data on circulating *S. pneumonia* serotypes were available for a limited proportion of the notified cases, however showed an overall reduction of those serotypes covered by commercialised pneumococcal conjugated vaccines, which is counterbalanced by an increase in serotypes contained in the recently introduced PPSV23 among the elderly and non-vaccine serotypes.

We observed an increasing trend in IPD notification rates at the country level, more than doubling from 2007 to 2017. Following the introduction of universal free of charge vaccination in Italy since 2007 [15], a renewed interest in IPD epidemiology may have resulted in a growing attention to the aetiological diagnosis of invasive disease [18], prompted case reporting and enhanced the surveillance system [6]. Yet, despite the progressive VC and existing evidence on vaccine-induced herd immunity from other European countries [19, 20], increasing IPD notification rates may be unexpected. Current IPD surveillance system in Italy was rolled out in 2007, yet IPD case notification remains voluntary and surveillance implementation has been progressively improving since then. Limited sensitivity of the current surveillance system has been suggested by a recent Italian study [21] and may be postulated comparing Italian and European data. In 2017, IPD notification rate in Italy was less than half than the EU/EEA average rate (6.23 per 100 000 in 2016, latest data available) [22]. With this in mind, it may be difficult for such a surveillance system to reliably distinguish between genuine changes in the disease epidemiology and possible surveillance artefacts.

Such observations become even more relevant when assessing IPD notification rates at the sub-national level, as our findings showed a marked North-South geographical gradient throughout the entire study period. While we may not exclude this to be the result of genuine variations in *S. pneumoniae* epidemiology across the country, a previous study comparing two different regions of Italy (one in the north and one in the south) suggested differences in case ascertainment and reporting to be the likely explanations for the observed divergence in case notification [23]. In Italy, healthcare management decentralisation includes disease monitoring activities and differences in local implementation of surveillance systems may influence comprehensiveness and sensitivity in certain areas of the country. Furthermore, the North-South gradient is not uniquely connected to IPD but characterises other communicable diseases surveillance in the country, such as Legionnaires' disease [24] or measles, for which no geographic pattern is expected to occur [25, 26].

While IPD notification rate increased in almost all the regions over the study period, likely attributable to a generalised increase of surveillance sensitivity, our analysis pointed towards the identification of three subgroups of regions, roughly corresponding to the northern, central and southern areas of Italy. Using the 1 per 100 000 rate threshold, as reported elsewhere [17], as an indirect proxy for surveillance performance, northern regions reported above the threshold throughout the study period, with rates consistent with EU/EEA values [27]. Central regions presented with increasing rates reaching above the threshold in recent years, suggesting a progressive improvement of the local surveillance systems. Southern regions showed minimal changes over time, reporting rates consistently below the threshold [28, 29].

We complemented trends analysis with the additional parameter of relative proportion of IPD reported cases by age group, in the attempt to adjust for increasing surveillance intensity and assuming no underlying systematic age-specific changes in physicians' ascertainment and reporting practices. Our findings showed a marked and concordant increasing trend for IPD notifications among individuals aged above 64 years, as compared to all other age groups. Similar increases in population aged above 64 years were reported in other European countries [30–32]. Our findings, alongside recent economic considerations [33] further support the introduction of a pneumococcal vaccination strategy targeting individuals aged above 64 years in Italy included in the latest NPVP released in 2017 [6]. Despite the plan ambitiously set a 75% target VC to be achieved by 2019, available data indicate much lower coverage level achieved so far. Most importantly, we could not find nationwide VC data among adults. This is an important gap to be addressed rapidly following the introduction of older adults vaccination in the country.

When considering children, the analysis of rates and relative case proportion by age resulted in discordant trends, with increasing age-specific rates and decreasing proportion of IPD cases in the same age groups. We largely attributed the notification rate pattern to surveillance artefacts due to increasing surveillance sensitivity with a baseline high level of underreporting [34]. Interestingly, when assessing IPD notification trends in selected northern regions, decreasing rates were observed over time, suggesting how better performing surveillance systems were able to detect vaccine-induced epidemiological changes [28, 29]. The reported serotypes distribution analysis further supported this view, with IPD cases due to serotypes contained in PCV13 decreasing over time, at least among children (see online Supplementary materials). Taken altogether, we interpreted these findings as signals of the impact of vaccination programmes among those birth cohorts targeted with universal routine vaccination since 2007 [27, 35]. A decrease in IPD cases among children in Italy would also be consistent with the reported high VC among infants in the country. Despite a certain degree of sub-national variability and some gaps in the data, VC increased over time and showed an upward peak in 2017, following the highly debated law on mandatory vaccination in Italy rolled out earlier that year [36].

Pneumococcal vaccines are available in several formulations, covering for discreet sets of serotypes [35, 36]. Continuous monitoring of circulating serotypes is of paramount importance to assess the impact of vaccination interventions, vaccine-induced serotypes replacement dynamics and to inform future vaccination strategies [37]. In Italy, PCV7 has been progressively introduced, since 2003, and its use was exclusive until 2009. From July 2010, PCV7 was replaced by PCV10 and PCV13 in some regions and later that year PCV13 was introduced into national pneumococcal vaccination programme [38]. Both vaccines have been shown to induce a herd protection effect [12, 39, 40] and to impact on serotypes circulation dynamics [39, 41–43]. Despite its relevance, microbiological surveillance of pneumococcal serotypes in Italy is limited to a fraction, albeit increasing over time, of the reported cases.

Available molecular data showed a marked decrease of IPD cases due to vaccine-related serotypes following the introduction of PCV7 in 2007, of PCV10 in 2009 and of PCV13 in 2010. Yet the proportion of cases due to non-PCV serotypes potentially preventable by PPSV23, namely 8, 12FF and 22F, have increased over time, alongside that of those not included in any available vaccine. This replacement phenomenon is well described in other European countries, such as UK, Denmark and France [39, 41–45] and deserves careful monitoring, even more so in the scenario of a progressive coverage of PPSV23 vaccination foreseen by the current NPVP.

Our study highlighted the existing challenges in describing accurately IPD epidemiology in Italy. The main issue we encountered was the incompleteness of the available data and data gaps. For example, VC data were not available for certain age groups and for all regions during the study period or age-specific notification data were not available by region. Although we collected and collated data on IPD epidemiology from different sources over a long-time frame, data linkage was technically not possible. These limitations prevented us from performing more advanced analysis to further explore IPD epidemiology or to correct for relevant confounding factors. In addition, while changes in case definition and data collection protocols at the national level are well documented, implementation coverage and compliance may have varied across the country and over time.

Finally, due to the limitations listed above, the representativeness of the gathered data may have been suboptimal and failed to provide an accurate picture of the IPD surveillance system and genuine changes in disease epidemiology across the entire country.

In conclusion, important limitations of IPD surveillance in Italy hamper its utility in assessing disease epidemiology in the country. Improved surveillance sensitivity over time makes it difficult to discern true changes from artefacts. Yet, despite weaknesses, there were indirect signals of a positive impact of vaccination programmes on the burden of disease among children. Available data also confirmed the need to implement vaccination among older adults as advocated by the NPVP. However, to ensure effective, timely and accurate assessment of the impact of vaccination policies, innovations and targets brought about by the 2017 NPVP, further investments would be warranted to improve coverage and completeness of the IPD surveillance system in Italy. These would include upgrading the system infrastructure, ensuring data sources inter-operability, optimizing reporting protocols and training of healthcare staff engaged in IPD cases diagnosis and reporting.

## Supplementary material

For supplementary material accompanying this paper visit http://dx.doi.org/10.1017/S0950268820001077. click here to view supplementary material
