# Supplementary material for: Impact of vaccination on invasive pneumococcal disease in Italy 2007–2017: surveillance challenges and epidemiological changes
Source: Epidemiol Infect. 2020 May 18;148:e187. doi: 10.1017/S0950268820001077 (PMC7482190; doi:10.1017/S0950268820001077)
Supplement: Supplementary file 1 [file S0950268820001077sup001.docx]

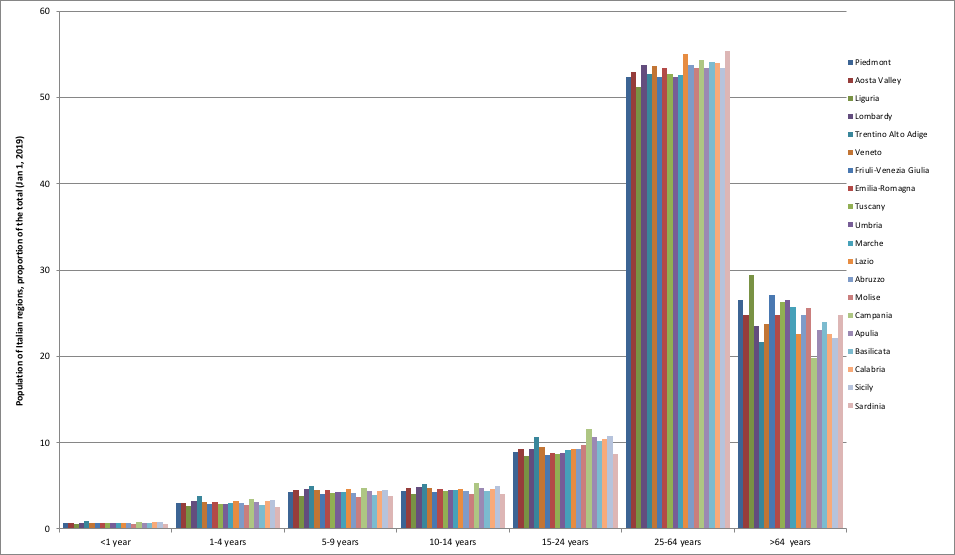


Figure 5: Population of Italian regions in all the age groups considered for the analysis (<1, 1-4, 5-9. 10-14, 15-24, 25-64 and >64 years)


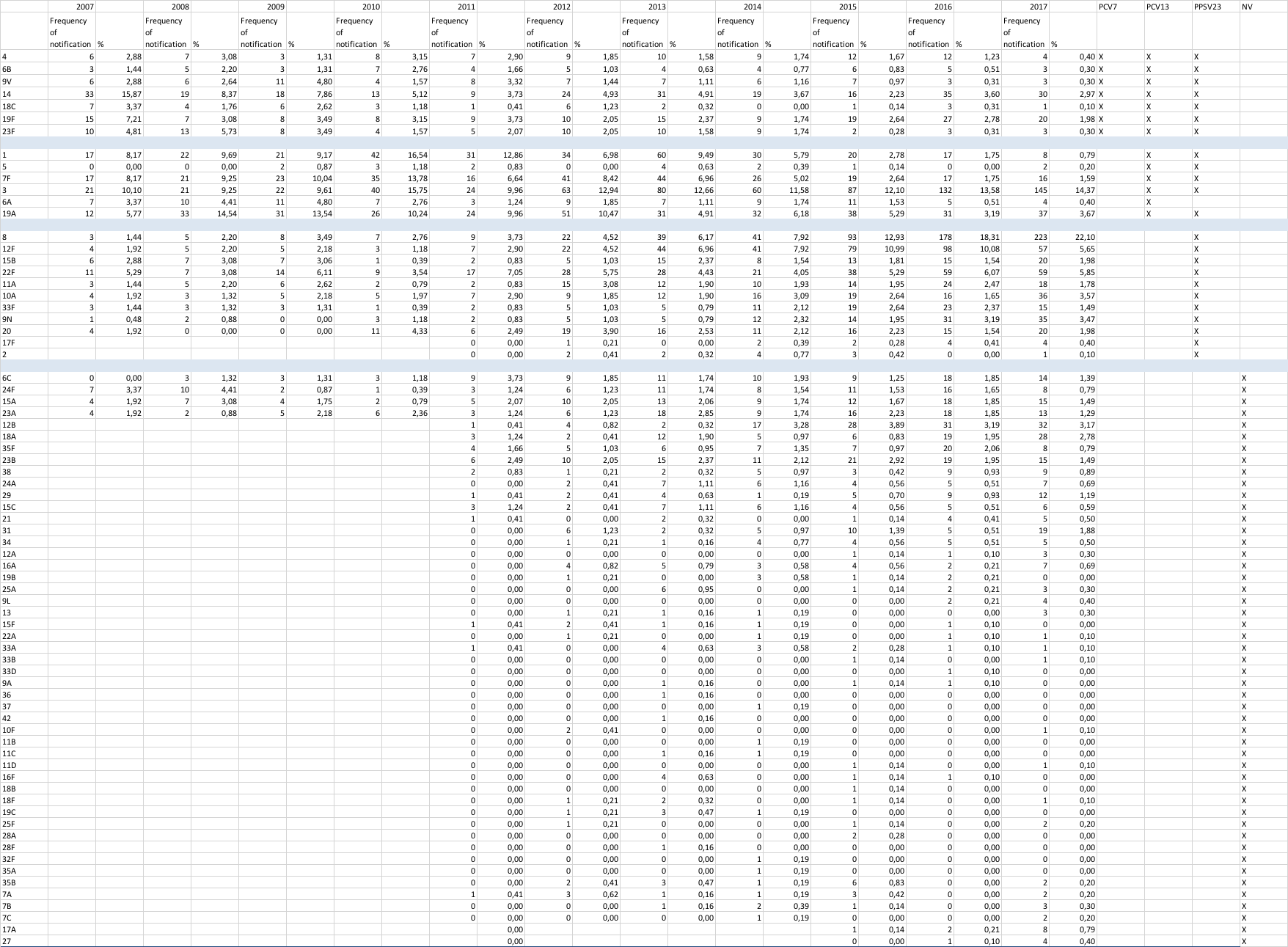


Table 2: Frequency of notification and % of total cases notified/year per single serotype in general population, Italy, 2007-2017


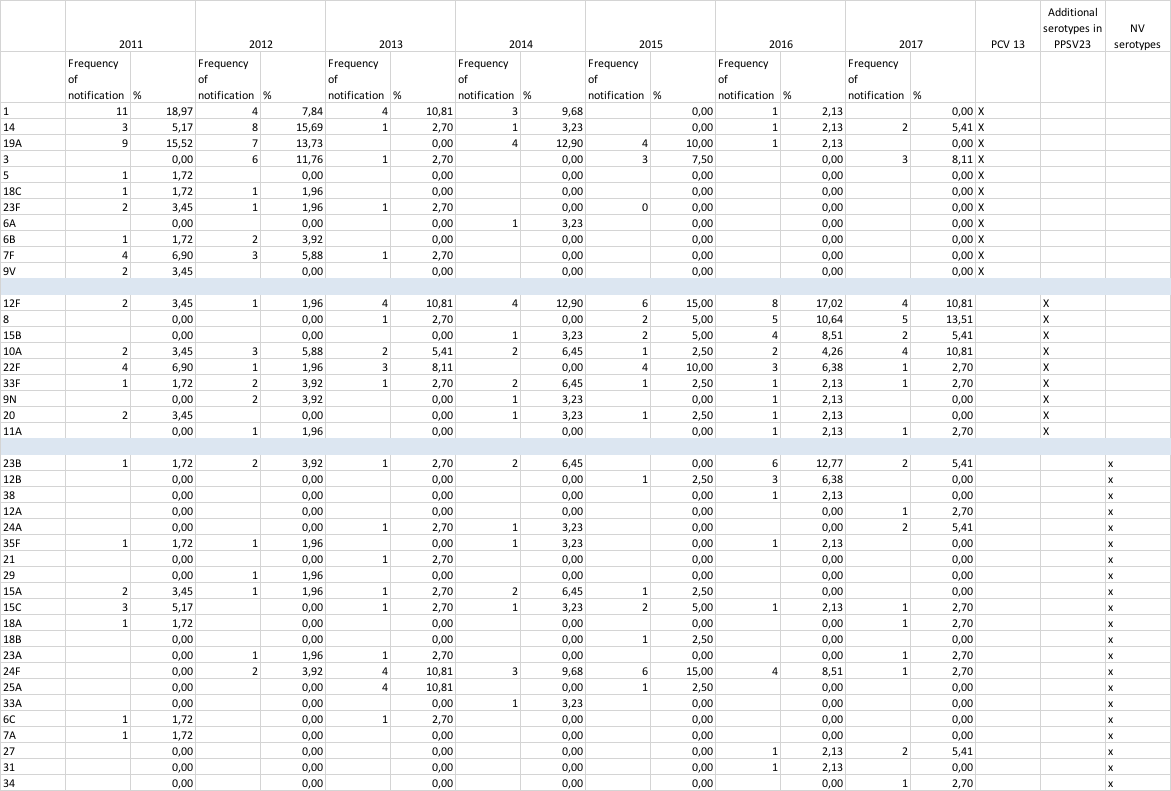


Table 3: Frequency of notification and % of total cases notified/year per single serotype in infants and children aged 4 years or less, Italy, 2011-2017


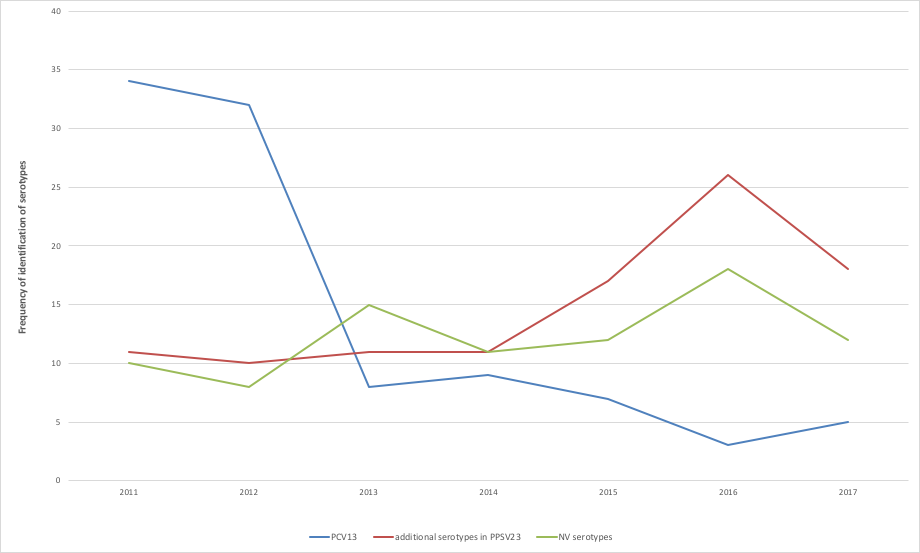


Figure 6: Frequency of identification of pneumococcal serotypes by vaccine formulation in infants and children aged 4 years or less, Italy, 2011-2017.


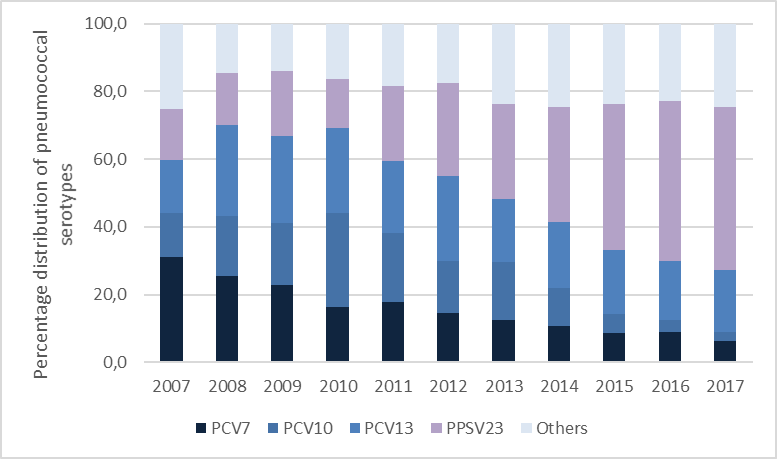


Figure 7: Distribution of pneumococcal serotypes by vaccine formulation, Italy, 2007-2017
